# Calcium-sensor proteins but not bicarbonate ion activate retinal photoreceptor membrane guanylyl cyclase in photoreceptors

**DOI:** 10.3389/fnmol.2024.1509366

**Published:** 2024-12-09

**Authors:** Igor V. Peshenko, Elena V. Olshevskaya, Alexander M. Dizhoor

**Affiliations:** ^1^Pennsylvania College of Optometry, Salus at Drexel University, Elkins Park, PA, United States; ^2^Department of Neurobiology and Anatomy, Drexel University, Philadelphia, PA, United States

**Keywords:** guanylyl (guanylate) cyclase, retina, photoreceptors, bicarbonate, phototransduction

## Abstract

Retinal membrane guanylyl cyclase (RetGC), regulated by guanylyl cyclase activating proteins (GCAPs) via negative calcium-feedback, is one of the most critically important enzymes in vertebrate rod and cone physiology, enabling their sensitivity to light. It was also reported that, similarly to olfactory receptor guanylyl cyclase, bicarbonate anion directly stimulates RetGC activity in photoreceptors as a novel phototransduction-linked regulating factor. We directly tested whether or not RetGC is a bicarbonate-activated enzyme using recombinant human RetGC expressed in HEK293 cells and the native RetGC in mouse retinas. Whereas RetGC in all cases was activated by GCAPs, we found no evidence indicating that bicarbonate can produce direct stimulating effect on RetGC catalytic activity, either basal or GCAP-activated, even at concentrations as high as 100 mM. Instead, near-physiological concentrations of bicarbonate only slightly reduced RetGC activity, whereas concentrations substantially exceeding physiological levels caused a more pronounced reduction of RetGC activity measured in mouse retinas. Our results argue that photoreceptor guanylyl cyclase is not a bicarbonate-stimulated enzyme and rule out the possibility that effects of bicarbonate on photoreceptor physiology are mediated by a direct stimulation of retinal guanylyl cyclase by HCO_3_^−^.

## Introduction

Vertebrate rods and cones respond to light by changing the permeability of cyclic nucleotide-gated cation channels in the plasma membrane of their outer segments, which depends on the relative rates of cGMP synthesis and decay between light and dark (Pugh et al., 1999). Retinal guanylyl cyclase (RetGC) produces cGMP to open the channels, whereas Gt-activated phosphodiesterase 6 hydrolyzes cGMP in the light to close the channels, thus causing hyperpolarization of the photoreceptor membrane and interrupting the tonic release of the neuromediator glutamate from the synaptic terminus of the cell (reviewed in Pugh et al., 1997; Koch and Dell'Orco, 2013, 2015; Dizhoor and Peshenko, 2021). Two RetGC isozymes are expressed in a vertebrate retina. RetGC1 (Dizhoor et al., 1994; Yang et al., 1995), also referred to as GG-E or ROS-GC1 (reviewed in Koch and Dell'Orco, 2013, 2015; Dizhoor and Peshenko, 2021), coded by *GUCY2D* gene in the human genome or *Gucy2e* gene in rodents (Yang et al., 1996), is the main RetGC isozyme producing cGMP as a messenger of photoransduction in vertebrate rod and cone outer segments, whereas rods also express as ancillary isozyme RetGC2, or GC-F (*GUCY2F*, *Gucy2F*) (Lowe et al., 1995; Yang et al., 1995, 1996; Yang and Garbers, 1997; Baehr et al., 2007; Olshevskaya et al., 2012). Ca^2+^/Mg^2+^-sensor proteins, GCAPs, regulate the enzymatic activity of RetGC isozymes in the outer segments of photoreceptors via negative Ca^2+^-feedback: Mg^2+^GCAPs stimulate the cyclase activity in photoreceptor outer segments after illumination to enable fast reopening of the closed channels and the recovery from the light-induced hyperpolarization, whereas Ca^2+^GCAPs in the dark decelerate it to maintain a small fraction of cGMP gated channels in the open state to provide partial depolarization of dark adapted rods and cones (reviewed in Dizhoor and Peshenko, 2021; Koch and Dell'Orco, 2013, 2015; Lamb and Hunt, 2018; Lim et al., 2014). It was also reported (Duda et al., 2015, 2016; Makino et al., 2019) that, similarly to the bicarbonate-activated olfactory receptor guanylyl cyclase GC-D (Guo et al., 2009; Sun et al., 2009), RetGC1 is a bicarbonate-activated enzyme, declaring that bicarbonate ion is a novel phototransduction-linked RetGC regulating factor shaping the photoresponse (Geva et al., 2022). In this study, we evaluated the hypothesis that RetGC is a bicarbonate-activated enzyme by directly testing its potential effects on the basal and GCAP-stimulated activity of RetGC.

## Materials and methods

### Animals

All experiments involving animals were conducted in accordance with the Public Health Service guidelines and approved by the Institutional Animal Care and Use Committee of Salus University (presently merged with Drexel University). C57Bl6 mouse strain was acquired from Jackson Laboratory. Transgenic mouse models used in this study were kindly provided by other investigators: RetGC2^−/−^ (Baehr et al., 2007) by Dr. Wolfgang Baehr (University of Utah) and GCAPs^−/−^ (Mendez et al., 2001) by Dr. Jeannie Chen (University of Southern California). All transgenic mouse lines were made congenic with the C57B6 background by back-crossing to C57Bl6 for over 10 generations prior to conducting the experiments. Male and female mice were used in the study non-discriminatory, without segregating the data by the sexes.

### GCAP1 expression and purification

N-myristoylated bovine or murine GCAP1 for the *in vitro* assays was expressed from pET11d vector in a BLR(DE3) *E. coli* strain (both originated from Novagen/Calbiochem) harboring a plasmid coding for a yeast N-myristoyl transferase and purified by butyl-Sepharose and Sephacryl S-100 size-exclusion chromatography using previously described in detail procedures (Peshenko and Dizhoor, 2006; Peshenko et al., 2008, 2011, 2012, 2016). The purity of GCAP1 preparations estimated by SDS gel electrophoresis was ≥90%.

### RetGC1 expression

Human recombinant RetGC1 was expressed from a modified Invitrogen pRCCMV vector in HEK293 cells transfected using calcium phosphate precipitation method, and the membrane fraction containing the expressed RetGC1 was isolated by centrifugation as previously described (Peshenko and Dizhoor, 2004).

### Guanylyl cyclase activity assays

**Figure 1:**
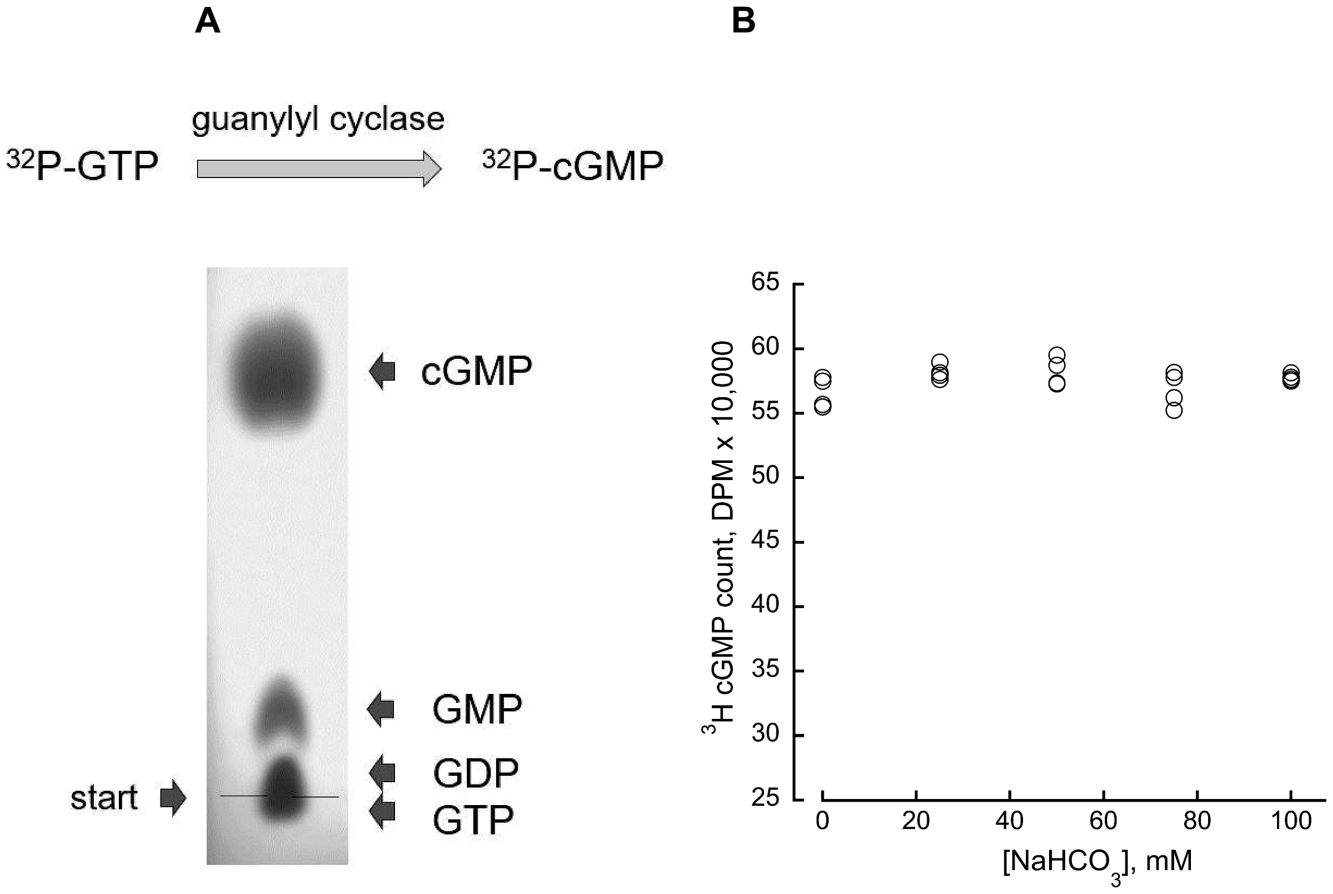
Direct measurement of guanylyl cyclase enzymatic activity. **(A)** (*Top*) the assay directly determines [*α*-^32^P] GTP conversion to [^32^P]3′:5’ cGMP. (*Bottom*) TLC chromatography on PEI cellulose-F plate isolates the product of the reaction (see Materials and Methods); an example of the cGMP isolation from a 5 μL mixture containing 1 mM GMP, 1 mM GDP, 1 mM GTP, and 3 mM 3′:5’ cGMP, on PEI-F plate developed in 0.2 M LiCl and photographed under UV illumination. **(B)** Bicarbonate does not affect cGMP recovery from the assay mixture by TLC; 5-μl aliquots from a standard assay mixture without added RetGC, containing 4 mM [^3^H]cGMP as an internal standard and varying concentrations of NaHCO_3_, were separated by TLC. The [^3^H]cGMP radioactivity eluted from the cGMP spot on the chromatogram was counted by liquid scintillation; the mean average ± SD (*N* = 4) for 0, 25, 50, 75 and 100 mM NaHCO_3_ was 56,615 ± 1,183, 58,175 ± 570; 58,219 ± 1,090 and 56,852 ± 1,362; 5,775 ± 273, respectively.

The RetGC activity was assayed as previously described in detail (Peshenko and Dizhoor, 2004) with minor modification described previously (Peshenko et al., 2016). In brief, the assay mixture (25 μL) containing HEK293 membranes, 30 mM Mops–KOH (pH 7.2), 60 mM KCl, 4 mM NaCl, 1 mM DTT, 2 mM EGTA, 10 mM Mg^2+^, 0.3 mM ATP, 4 mM cGMP, 1 mM GTP, and 1 μCi of [*α*-^32^P]GTP (Perkin Elmer), 100 μM zaprinast and dipyridamole was incubated at 30°C for 40 min, and the reaction was stopped by heat inactivation at 95°C for 2 min. The resultant [^32^P]cGMP product was separated by thin-layer chromatography (TLC) using fluorescently backed polyethyleneimine (PEI) cellulose plates (Merck) developed in 0.2 M LiCl (Figure 1 and Table 1). The cGMP spots cut from the plate were eluted with 2 M LiCl in scintillation vials, and the radioactivity was measured using liquid scintillation. Mouse retinas for RetGC activity measurements were excised from dark-adapted ~4 weeks-old mice under infrared illumination (Kodak number 11 infrared filters) using a dissecting microscope fitted with an Excalibur infrared goggles. The excised retinas were wrapped in aluminum foil, frozen in liquid nitrogen, and stored at −70°C prior to using them in RetGC activity assays. The assays were also conducted under infrared illumination using night vision optics. The retinas were homogenized in 75 μL/per retina of 60 mM Mops–KOH (pH 7.2), 120 mM KCl, 8 mM NaCl, 2 mM DTT, 4 mM EGTA, 5 mM Mg^2+^, 0.4 mM ATP, 200 μM zaprinast and dipyridamole, 20 mM creatine phosphate and 0.5 unit per retina of creatine phosphokinase (Sigma–Aldrich), and 12.5 μL of the homogenate was added in the assay mixture (total assay volume 25 μL). The reaction was performed as described above except that the incubation time for the retinal samples was 12 min, during which period the time-course of the reaction remained linear. Creatine phosphate/creatine phosphokinase was used as GTP regenerating system to maintain the linear time-course of the reaction during the assay, counteracting [*α*-^32^P]GTP hydrolysis to [α-^32^P]GDP in retinal homogenates by the mitochondrial membranes (most of which are removed from the HEK membrane preparations and it is therefore not required to use regeneration system to maintain the linear time-course in the case with HEK-expressed recombinant RetGC). The presence of the regenerating system does not affect the subsequent TLC separation of the products of the reaction. The assay mixture also contained ~0.1 μCi of [^3^H]cGMP (Perkin Elmer) as the internal standard to verify the lack of the cGMP hydrolysis by retinal phosphodiesterase in the assay. Data fitting was performed using a Synergy KaleidaGraph software. Notably, separation of cGMP by TLC was not significantly affected by the presence of sodium bicarbonate in the sample (Figure 1). It is also important to note that addition of freshly prepared NaHCO_3_ stock solutions used in all experiments altered the pH in the reaction mixture buffered by 30 mM MPOS–KOH insignificantly, from pH7.2 to 7.45 (directly measured with a pH-electrode) at the maximal used NaHCO_3_ concentration of 100 mM. Such a small, by 0.3, increase of pH, would still be within the optimal pH range for the cyclase activity and could only elevate RetGC activity in our assays by a small fraction, ~8%, compared to the pH7.2. The actual maximal change of the pH at 100 mM NaHCO_3_ did not reach 0.3 pH unit and only increased the pH by 0.08–0.18 at 25–50 mM NaHCO_3_. Therefore, we did not consider NaHCO_3_ as a factor that could substantially alter RetGC activity via pH change on the reaction mix.

**Table 1:** Typical example of the radioactivity counting in the [^32^P] cGMP spot isolated by TLC from a 5-μl aliquot of the standard guanylyl cyclase assay mixture (Materials and Methods).

Sample content	[^32^P]cGMP isolated by TLC, DPM
[^32^P]GTP, no RetGC1	60 ± 2
[^32^P]GTP, RetGC1, Mg^2+^GCAP1	54,000 ± 2000

The total [^32^P] radioactivity in a 5-μl aliquot of the mixture directly added to 10 mL of scintillation cocktail in this example was 937,000 ± 20,000 DPM in both cases.

## Results

### Bicarbonate does not stimulate the enzymatic activity of recombinant RetGC1

**Figure 2:**
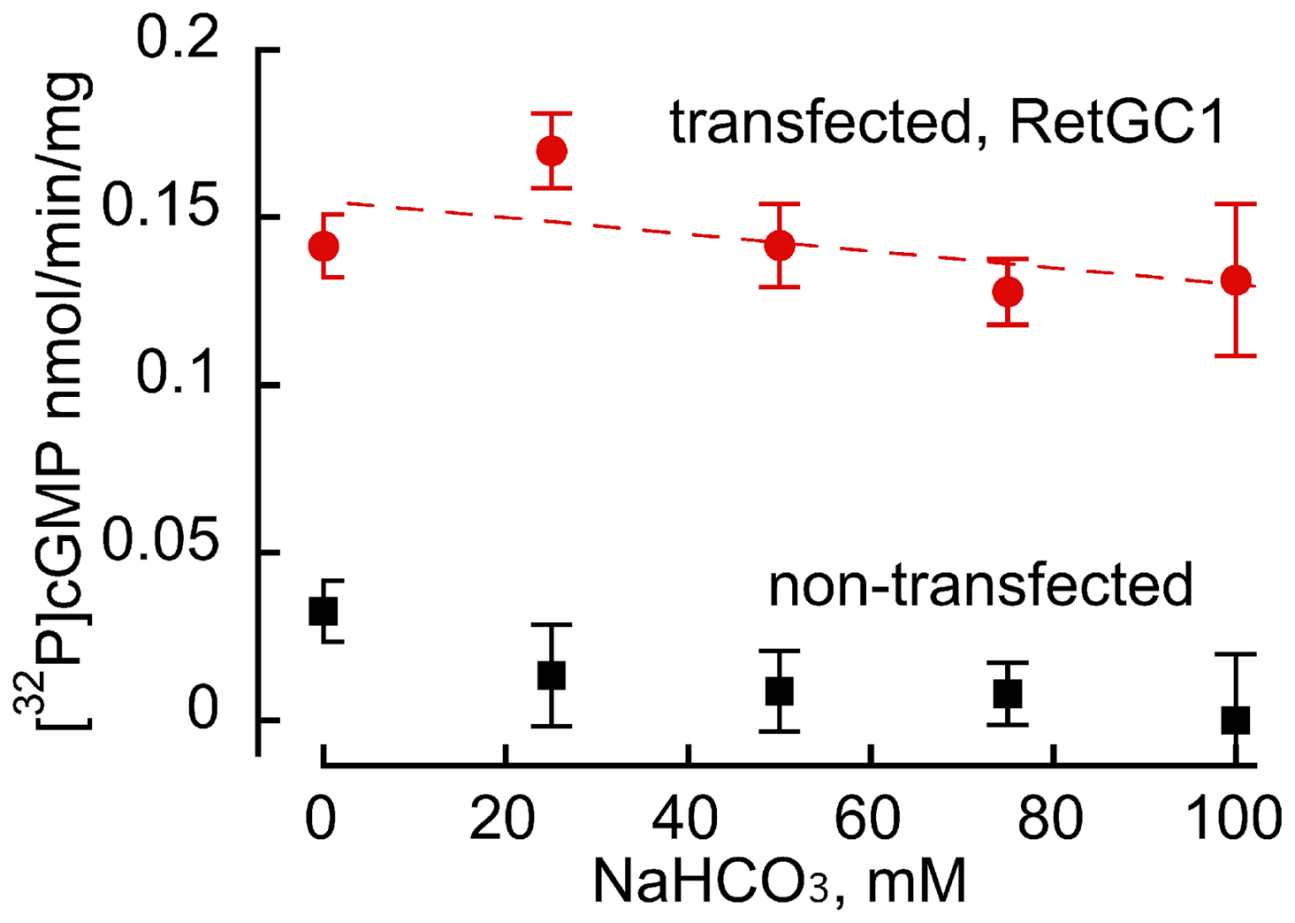
Bicarbonate does not stimulate basal activity of recombinant RetGC1 in the absence of GCAP. Guanylyl cyclase activity (nmol cGMP/min/mg membrane protein) was assayed in membrane fraction of HEK293 cells transfected with wild-type RetGC1 cDNA (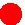) or non-transfected HEK293 membranes (■) in the presence of 10 mM MgCl_2_, 2 mM EGTA and various concentrations of bicarbonate: 0, 25, 50, 75 and 100 mM, with the respective mean ± SD (*N* = 3 replicates for each) values of 0.142 ± 0.004, 0.169 ± 0.016, 0.142 ± 0.0043, 0.128 ± 0.005, and 0.131 ± 0.048 nmol cGMP/min/mg; there was no statistically significant change of the activity by NaHCO_3_ at any of the tested concentrations (*t*-test *p* > 0.12).

**Figure 3:**
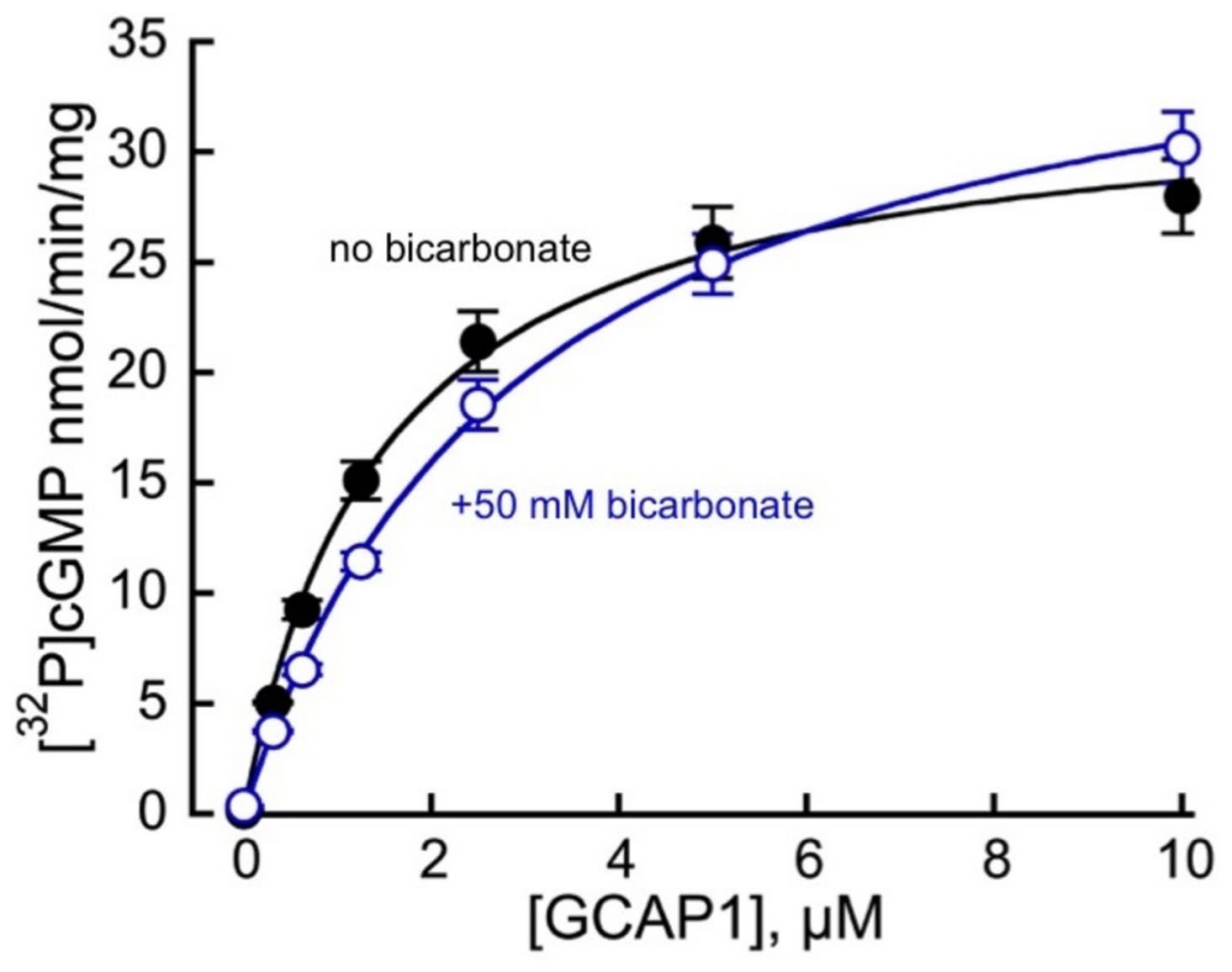
Mg^2+^GCAP1-stimulated guanylyl cyclase activity of recombinant RetGC1 in the presence (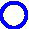) and in the absence (●) of 50 mM NaHCO_3_ at the indicated concentrations of purified myristoylated bovine GCAP1. The respective mean ± SD (*N* = 4) values for the activities measured at 0, 0.312, 0.625, 1.25, 2.5, 5, and 10 μM GCAP1 were 0.14 ± 0.03, 5.06 ± 0.07, 9.26 ± 0.85, 15.1 ± 1.7, 21.4 ± 2.7, 25.9 ± 3.2 and 28 ± 3.4 nmol cGMP/min/mg membrane protein in the absence versus 0.36 ± 0.03, 3.7 ± 0.08, 6.5 ± 0.5, 11.4 ± 0.8, 19 ± 2.2, 25 ± 2.7 and 30 ± 3.2 nmol cGMP/min/mg membrane protein in the presence of 50 mM bicarbonate. When the data are fitted assuming a hyperbolic function, A = A_max_ × [GCAP]/(K_1/2_ + [GCAP]), the respective K_1/2_ value (mean ± SD) in the presence of bicarbonate appeared marginally increased (2.9 ± 0.21 μM GCAP1 versus 1.47 ± 0.05 μM; t-test *p* = 0.00091), whereas A_max_ was not significantly different (*p* = 0.21).

HEK293 membranes containing recombinant RetGC1 display very low activity in the absence of GCAP1 and a dramatic stimulation of the activity by GCAP (Figures 2, 3). Whereas in Duda et al. (2015, 2016) publications bicarbonate stimulated RetGC, nearly as efficiently as GCAP, no such stimulating effect on the basal activity of the recombinant RetGC1 expressed in HEK293 cells was observed in the presence of 0–100 mM bicarbonate (Figure 2), so, the basal activity of the recombinant enzyme remained at near-zero levels. Whereas Mg^2+^GCAP1 activated recombinant RetGC1 expressed in HEK293 cells more than 100-fold, there was no obvious additional increase in GCAP1-stimulated RetGC1 activity in the presence of bicarbonate (Figure 3). Instead, only a minor right-shift in the GCAP dose-dependence in activating RetGC1 was observed in the presence of 50 mM NaHCO_3_: the 1.5 μM GCAP1 concentration presenting the K_1/2_ in a hyperbolic fit increased to 2.9 μM GCAP1 in the presence of 50 mM NaHCO_3_ (Figure 3), indicating a possible slight decrease in the efficiency of GCAP1 binding to the membranes containing the recombinant RetGC, resulting from the high concentration of bicarbonate. We cannot exclude that this, quite marginal, change, could be a non-specific effect of excessive salt concentration in the reaction created by additional 50 mM NaHCO_3_. Although the effect of salt was not systematically studied, a similar small right-shift (from 1.5 to 2.0 μM for the K_1/2_) could also be observed in the presence of additional 50 mM NaCl instead of bicarbonate in a pilot measurement (not shown).

### Bicarbonate does not stimulate native retinal guanylyl cyclase in mouse retinas

**Figure 4:**
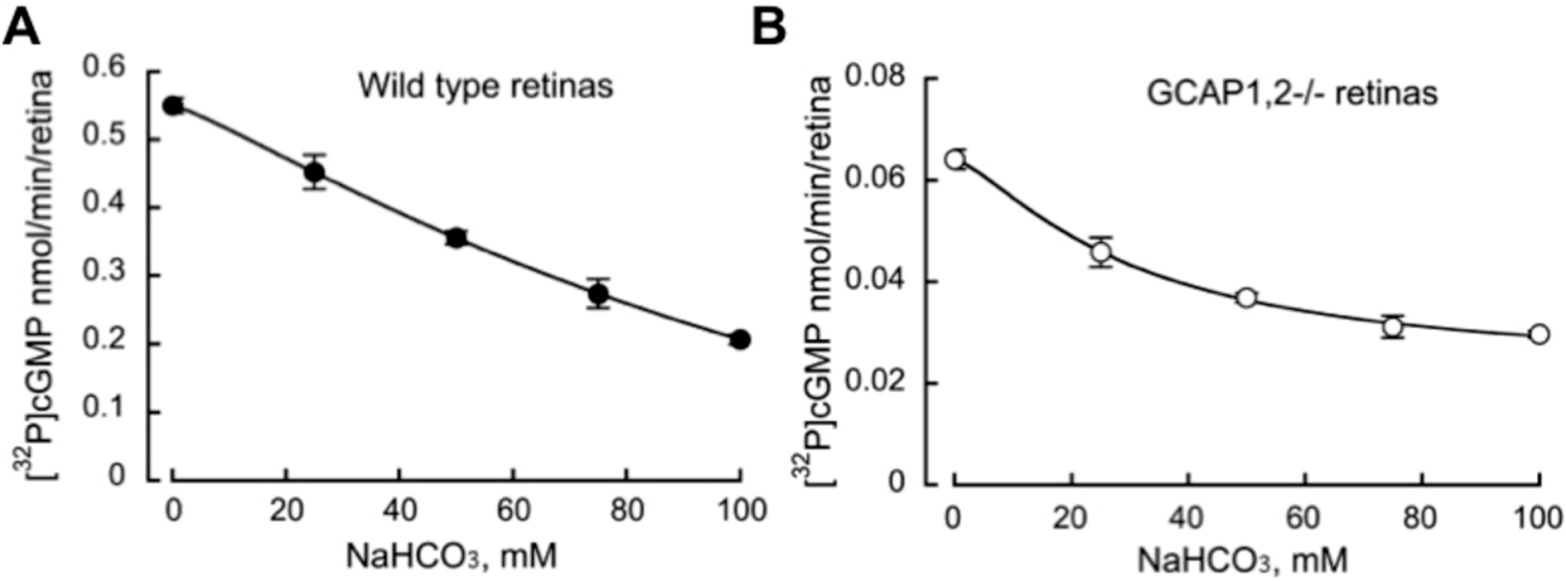
Bicarbonate decreases the total native guanylyl cyclase activity of RetGC1 and RetGC2 in the retinas of wild type **(A)** and GCAP1,2^−/−^ mice. **(B)** RetGC activity in homogenates (mean ± SD, *N* = 3) from the dark-adapted retinas was assayed under infrared illumination in the presence of 1 mM free Mg^2+^, 2 mM EGTA, and variable concentrations of sodium bicarbonate.

**Figure 5:**
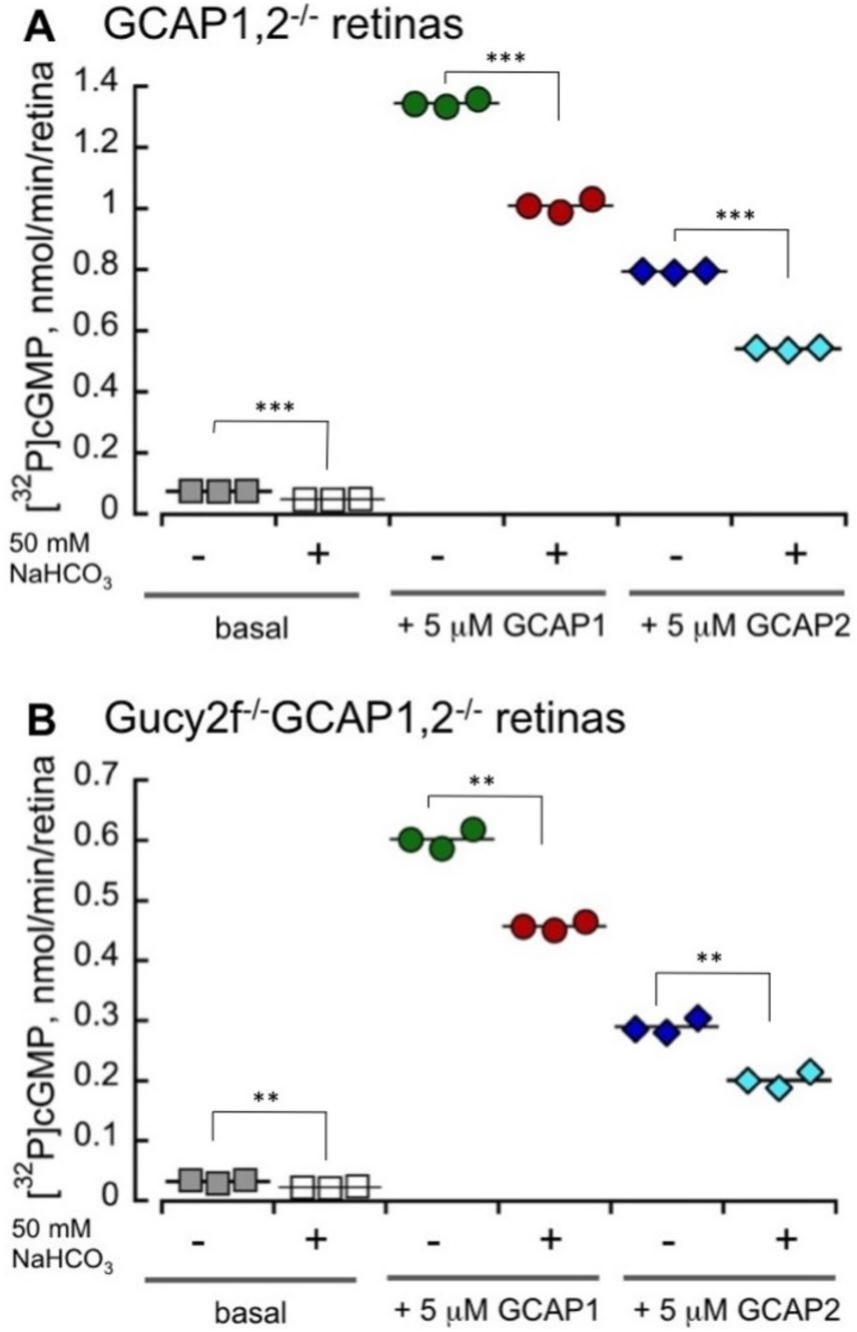
High concentrations of bicarbonate decrease both GCAP1 and GCAP2-stimulated activity of the total native RetGCs and the RetGC1 isozyme in mouse retinas. RetGC activity was measured in dark-adapted retinas from GCAPs−/− **(A)** and RetGC2−/-GCAPs−/− mice **(B)** reconstituted with 5 μM purified myristoylated recombinant murine GCAP1 or GCAP2 as described under *Materials and Methods*. The RetGC activity (mean ± SD, three repeats) was assayed in the dark under infrared illumination. ** *p* ≤ 0.01, ***p ≤ 0.001 (*t*-test). The assay mixtures contained 1 mM free Mg^2+^, 2 mM EGTA, and purified recombinant GCAP1 or GCAP2, as indicated, in the presence or absence of 50 mM sodium bicarbonate. The ‘basal activity’ assay contained bovine serum albumin instead of GCAPs.

Bicarbonate in our experiments not only failed to stimulate, but rather detectably reduced the total endogenous activity of RetGC measured in wild-type mouse retinas harboring both RetGC1 and RetGC2 endogenous isozymes, together with endogenous GCAP1 and GCAP2 (Peshenko et al., 2011; Olshevskaya et al., 2012) (Figure 4). The reduction of the total Mg^2+^GCAP-stimulated RetGC activity in wild type retinas by bicarbonate was concentration-dependent: the inhibitory effect was rather modest: less than 20% reduction of the activity was detected at 25 mM bicarbonate, which is considered to be within the normal physiological concentrations (Newman, 1991; Cowley et al., 2013; Sada et al., 2022). The reduction became more pronounced at higher concentrations of NaHCO_3_ exceeding the normal physiological levels (Figure 4A). Similarly, the integral basal catalytic activity of the native murine retinal guanylyl cyclase in the absence of GCAPs, measured in photoreceptor membranes extracted from GCAPs^−/−^ mouse retinas, was not stimulated by bicarbonate at physiological concentrations. Instead, the basal activity in the presence of bicarbonate was reduced (Figure 4B), similarly to the RetGC activity measured in the presence of endogenous GCAPs. Reconstitution with purified recombinant Mg^2+^GCAP1 or Mg^2+^GCAP2 resulted in respective 18- and 11-fold stimulation of the total RetGC activity (RetGC1 plus RetGC2) (Peshenko et al., 2011) in GCAPs−/− retina samples (Figure 5A) as well as the native RetGC1 activity, the only RetGC isozyme present in RetGC2^−/-^GCAPs^−/−^ retinas (Peshenko et al., 2011) (Figure 5B). In all these cases, bicarbonate at high concentration failed to additionally stimulate the cyclase activity, and instead even reduced it (Figures 5A,B).

## Discussion

Previous reports addressing the ability of bicarbonate to activate photoreceptor guanylyl cyclase contradicted each other. Whereas Sun et al. (2009) and Guo et al. (2009), presented evidence that HCO_3_^−^ was able to directly activate olfactory receptor guanylyl cyclase GC-D *in vitro* but not photoreceptor guanylyl cyclase, Duda et al. (2015, 2016) and Makino et al. (2019), reported direct activation of the photoreceptor cyclase by HCO_3_^−^ and declared that the activation of photoreceptor cyclase by HCO_3_^−^ resulted from specific binding of this anion to the cyclase. Our study, conducted using both the recombinant and the native forms of photoreceptor membrane guanylyl cyclase, rules out the possibility that RetGC is a bicarbonate-stimulated enzyme. In our experiments, utilizing bovine and murine GCAP-based regulation of the cyclase activity, bicarbonate did not stimulate either recombinant or native RetGC, whether in the presence or absence of its activators, GCAPs (Figures 3–5). The only detectable effect of HCO_3_^−^ was a moderate reduction of RetGC basal activity and GCAP-stimulated activity in mouse retinal homogenates at near-physiological concentrations and a more pronounced inhibition at higher-than-physiological concentrations, which according to the Duda et al. (2015, 2016) and Makino et al. (2019) publications were expected to produce a stimulating effect on the cyclase activity comparable with that of GCAP. The effect of the bicarbonate, if anything, was therefore contrary to what would be expected. The attenuation, however, was relatively small and could be, for example, merely a non-specific result of higher salt concentrations, potentially creating less than optimal conditions for the catalytic activity of the enzyme, or a potential effect of high concentrations of bicarbonate affecting membrane stability. Nonetheless, even the partial reduction of RetGC activity caused by NaHCO_3_ was contrary to what was reported by Duda et al. (2015, 2016).

It is not immediately apparent why the results of our study, along with the previous study by Sun et al. (2009) and Guo et al. (2009), are so much at variance with those reported by Duda et al. (2015, 2016). One possible reason is that in our study, we measured the catalytic activity of RetGC by directly quantifying the conversion of [*α*-^32^P]GTP to [^32^P]cGMP. This technique, a minor modification of the original method used by Koch and Stryer (1988), does not involve additional coupled or ligand-binding reactions, provides efficient separation of cGMP as the product of the RetGC catalysis (Figure 1, Table 1), and is not affected *per se* by the presence of NaHCO_3_ even at the highest concentrations used in our assays (Figure 1). In contrast, Duda and colleagues used an approach based on radioimmunoassay utilizing anti-cGMP antibody generated in their laboratory. We have not found detailed information in the literature describing how efficiently and specifically that particular antibody reacted with cGMP under various conditions. Therefore, we cannot exclude the possibility that both the binding affinity and/or the specificity of the antibody were affected by the high concentrations of bicarbonate in their assays, leading to the conclusion that bicarbonate affected the activity of the cyclase itself, rather than the detection of cGMP by the antibody.

Based on the assumption that RetGC is directly stimulated by HCO_3_^−^ (Duda et al., 2015, 2016; Makino et al., 2019). Geva et al. (2022) later reported a physiological study in which the effect of bicarbonate on rod photoresponse was interpreted as a result of direct stimulation of the cyclase activity. However, that study did not fully consider alternative interpretation, such as possible effects of changes in osmolality, fluid or ion co-transport with HCO_3_^−^ or that bicarbonate in their experiments could affect either directly or indirectly the intracellular levels of GTP as the substrate rather than changed the catalytic properties of the cyclase itself. For example, RetGC affinity for GTP as a substrate is much lower than the affinity of Gt protein for GTP as a co-factor required for light-dependent activation of PDE6 by Gt to produce photoresponse (Bennett and Dupont, 1985). The K_m,GTP_ of the cyclase lies within a submillimolar range (Dizhoor et al., 1994; Peshenko et al., 2011), which is close to the levels of GTP reported in amphibian photoreceptor outer segments (Biernbaum and Bownds, 1985). Therefore, an increase or decrease in the intracellular GTP, either directly or via modulation of ATP levels as a general physiological response to bicarbonate, could potentially affect the overall rate of cGMP production by the cyclase in photoreceptors without affecting Gt activation by rhodopsin, for example. This is merely one of the possibilities, and it is difficult at present to decisively identify the exact biochemical mechanisms leading to the effects of HCO_3_^−^ on rod photoresponses observed by Geva et al. (2022). Nonetheless, the results of our study strongly argue that RetGC is not a bicarbonate-activated enzyme, and that direct stimulation of RetGC catalytic activity by HCO_3_^−^ is not one of such mechanisms.

## Data Availability

The raw data supporting the conclusions of this article will be made available by the authors, without undue reservation.
